# Development of new microalgae-based sourdough “crostini”: functional effects of *Arthrospira platensis* (spirulina) addition

**DOI:** 10.1038/s41598-019-55840-1

**Published:** 2019-12-19

**Authors:** Alberto Niccolai, Manuel Venturi, Viola Galli, Niccolò Pini, Liliana Rodolfi, Natascia Biondi, Massimo D’Ottavio, Ana Paula Batista, Anabela Raymundo, Lisa Granchi, Mario R. Tredici

**Affiliations:** 1 Department of Agriculture, Food, Environment and Forestry (DAGRI), University of Florence, Florence, Italy; 2 FoodMicroTeam S.r.l, Florence, Italy; 3 Fotosintetica & Microbiologica S.r.l, Florence, Italy; 4 LEAF – Linking Landscape, Environment, Agriculture and Food, Instituto Superior de Agronomia, Universidade de Lisboa, Lisboa, Portugal

**Keywords:** Applied microbiology, Industrial microbiology

## Abstract

The aim of this work was to evaluate the influence of *Arthrospira platensis* F&M-C256 (spirulina) incorporation on the nutritional and functional properties of “crostini”, a leavened bakery product largely consumed in Italy and Europe. Sourdough was used as leavening and fermentation agent and three concentrations of *A. platensis* F&M-C256 were tested: 2%, 6% and 10% (w/w). Despite a lower volume increase compared to the control, the *A. platensis* F&M-C256 “crostini” doughs reached a technological appropriate volume after fermentation. At the end of fermentation, no significant differences in microorganisms concentrations were observed. *A. platensis* F&M-C256 “crostini” showed higher protein content compared to the control. Considering the European Commission Regulation on nutritional claims, “crostini” incorporated with 6% and 10% biomass can be claimed to be a *“source of protein”*. Six and ten percent *A. platensis* “crostini” also presented significantly higher antioxidant capacity and phenolics. A significantly lower value of *in vitro* dry matter and protein digestibility between *A. platensis* F&M-C256 “crostini” and the control was found. The overall acceptability decreased with increasing *A. platensis* F&M-C256 addition. The combination of spirulina biomass addition and the sourdough technology led to the development of a novel microalgae-based bakery product with nutritional and functional features.

## Introduction

Bakery products are a staple presence of the diet worldwide. This category includes different food products such as bread, cakes, pastries, biscuits and breakfast cereals. In Italy, many bakery goods are obtained through sourdough technique that is used both for artisanal and industrial productions^1–4^. Sourdough is typically defined as a mixture of water and flour fermented by lactic acid bacteria (LAB), primarily responsible for acidification, and yeasts, that promote the leavening process^5,6^. Sourdough LAB are mainly heterofermentative species belonging to *Lactobacillus*, while typical yeasts associated with this food matrix are *Saccharomyces cerevisiae*, *Candida milleri* and *Issatchenkia orientalis*^5^. The microbial populations are kept stable, with a LAB/yeast ratio between 1:10 and 1:100, and active through daily refreshments^2,4,7^. In addition to traditional products (e.g. typical breads and sweet leavened cakes), sourdough is being used for different baked goods: snacks, crackers, “grissini”, leading to the development of novel products. Indeed, the use of sourdough is recognized to improve rheological, sensory, technological and nutritional features of the final products, conferring peculiar and distinctive characteristics^8^. Sourdough fermentation lowers the glycaemic index by reducing starch digestibility, increases the bioavailability of mineral salts by reducing phytate content, and promotes proteolysis by activating endogenous cereal proteases and through LAB peptidases^9–13^. Some LAB strains can also produce bioactive molecules with nutraceutical properties such as amino acid derivatives (e.g. γ-amino butyric acid)^14^, bioactive peptides^15–17^, or exopolysaccharides with prebiotic potentialities^18,19^.

In order to further enhance bakery products overall quality, the addition of functional ingredients has been proposed: pseudo-cereals, legumes, sprouted cereals, wheat germ, fibers, microalgae, spices, herbs, etc.^14,20–24^. Indeed, new functional foods with a high nutritional value, consumable on a daily basis, are increasingly requested^25^.

The cyanobacterium *Arthrospira platensis*, often incorrectly reported as *Spirulina platensis*^26^ and known commercially as “spirulina”, can be used as food ingredient for the production of functional bakery products such as biscuits^20^ and bread^21^. Cyanobacteria, such as *A. platensis*, *Aphanizomenon flos-aquae* from Klamath Lake and *Nostoc sphaeroides* are rich in high biological value proteins, polyunsaturated fatty acids, carotenoids, vitamins, minerals, phenolic compounds and bioactive molecules^27^. In particular, *A. platensis* is considered a source of proteins (up to 70% of dry weight), iron, γ-linolenic acid, sulphated polysaccharides and phycocyanin^28^. *A. platensis* also shows various activities of pharmacological interest, such as antioxidant, immunomodulatory, hypolipidemic and anti-inflammatory activity^29–31^. An *in vitro* trial showed an absence of toxicity of *A. platensis* F&M-C256 aqueous and methanolic extracts against *Artemia salina* and a low toxicity level for the same aqueous extract against human dermal fibroblasts^32^.

*A. platensis* is considered a “safe food source” in several countries (e.g. Europe, Australia, the USA) and, having been used as food before May 1997, it is authorized in Europe for food consumption^33^. The safety of *A. platensis* for human consumption is supported by its long history of use as food component^34^. In Africa *A. platensis* is integrated into traditional preparations, called Dih’e^34,35^, and consumed from 10 to 40 g per person per day^36^. Despite the limited acceptance by consumers, several companies are starting to pay attention to the microalgae sector and the algae products market is expected to reach US$ 44.7 billion by 2023^37^. Examples of large companies that are investing in innovative algae-based food products are Corbion (https://www.corbion.com/algae-portfolio) and Dulcesol Group (http://en.dulcesol.com/). Although the emergence of spirulina-derived food products is increasing in the market, the launch of these products is not necessarily accompanied by thorough product development studies. A deeper comprehension of the complex interactions which take place in the different food matrixes and technological processes, as well as the deriving positive and negative effects on human health, should be considered in order to support a successful integration of this innovative ingredient in the diet, and the consumer acceptance in the long-term. Previous studies have shown that the addition of *A. platensis* biomass can promote significant impacts on a food product microstructure, nutritional and functional properties, namely in bakery products such as biscuits^20^ and pasta^21^. When dealing with fermented products it is also expected that the introduction of cyanobacterial biomass might impact the microorganisms performance and the final products properties. Some studies on spirulina-enriched bread have been published^38,39^, but to the best of our knowledge, no literature data on spirulina-based bakery product fermented by sourdough are available.

“Crostini” is a typical Italian leavened product, obtained by cooking and successive toasting to reach a humidity less than 10% on final product weight^40^. The aim of this work was to evaluate the influence of incorporating *A. platensis* F&M-C256 biomass on the nutritional and functional properties of “crostini” made by using sourdough. The effects of *A. platensis* F&M-C256 addition (2, 6 and 10% w/w) in the dough were evaluated through physicochemical, microbiological and sensory analyses.

## Materials and Methods

### *A. platensis* F&M-C256 biomass production

Biomass of *Arthrospira platensis* F&M-C256, a strain belonging to the culture collection of Fotosintetica & Microbiologica S.r.l. (F&M), Italy, was produced at Azienda Agricola Serenissima S.S. (Conche di Codevigo, Padova, Italy). The cyanobacterium was cultivated in Zarrouk medium^41^ in GWP^®^-II photobioreactors^42^ in semi-batch mode, harvested by filtration, and the biomass was washed with tap water to remove excess bicarbonate. The biomass was then dried at low temperature (33 °C) (North West Technology, Cuneo, Italy) for 20 h and the obtained flakes were stored at −20 °C until use. The biochemical composition of the biomass was determined as Abiusi *et al*.^43^, moisture and ash were analysed following ISTISAN protocols^44^ (Table 1).

**Table 1:** Biochemical composition of *A. platensis* F&M-C256 biomass used in the experiments. Data are expressed as % (w/w) of algal powder. Results are expressed as average ± standard deviation (n = 3).

	Moisture	Ash	Lipid	Protein	Carbohydrate
*A. platensis* F&M-C256	7.7 ± 0.1	5.8 ± 0.1	6.7 ± 0.002	67.3 ± 0.01	12.5 ± 0.2

### Lactic acid bacteria and yeast growth conditions

*Lactobacillus farciminis* DZB19 and *Saccharomyces cerevisiae* LV8 strains isolated from Italian sourdoughs and belonging to the collection of the Department of Agriculture, Food, Environment and Forestry of the University of Florence (Italy), were used in this study. *L. farciminis* was routinely propagated for 24 h at 30 °C in MR3i medium^45^, *S. cerevisiae* LV8 strain was aerobically cultured at 30 °C in MYPG, a medium containing (in g/L): malt extract 5, yeast extract 3, meat extract 5 and glucose 10. Cells were recovered by centrifugation (5,000 × g for 20 min), successively washed in physiological solution and used for the preparation of the sourdoughs.

### “Crostini” preparation

Sourdough was prepared inoculating *L. farciminis* DZB19 at the initial concentration of 7 log CFU/g and *S. cerevisiae* at 6 log CFU/g in a dough made with 190 g Type “00” wheat flour and 110 mL water, with a dough yield [DY = (amount of flour + amount of water) x100/(amount of flour]) of 158. The sourdough was fermented for 18 h at 30 °C in a proofing room, before being used for “crostini” preparation.

The recipe of “crostini” is reported in Table 2. *A. platensis* F&M-C256 biomass was added at different percentages by replacing total flour: 2% (Ap 2 “crostini”), 6% (Ap 6 “crostini”) and 10% (Ap 10 “crostini”). A control, without microalgae incorporation was also prepared and analysed. The ingredients were mixed in a twin arm mixer (Bernardi, Italy) at room temperature for 10 min at 50 rpm mixing rate. After 25 min of proofing at 25 °C, the doughs were divided into batches of 300 g, to obtain molds of 25 cm length and 1.8 cm diameter. Afterwards, the molded doughs were fermented at 30 °C for 2 h and baked at 160 °C for 11 min. Finally, the “crostini”, were obtained by cutting the molds in pieces of 1.1 cm thickness and then toasted at 140 °C for 14 min. After cooling, sample “crostini” were stored at room temperature in hermetic containers, protected from light. Some of the “crostini” batches were immediately crushed to powder and frozen to be used for chemical analysis, antioxidant capacity, and *in vitro* digestibility.

**Table 2:** “Crostini” recipes (%, w/w). Control (without *A. platensis* F&M-C256 incorporation) (C) and “crostini” enriched with 2% (Ap 2), 6% (Ap 6) and 10% (Ap 10) (w/w) *A. platensis* F&M-C256 biomass.

Ingredients	C	Ap 2	Ap 6	Ap 10
Sourdough	25	25	25	25
Wheat flour	47	45	41	37
Water	17	17	17	17
Extra virgin olive oil	10	10	10	10
Salt	1	1	1	1
*A. platensis* F&M-C256	0	2	6	10

### Dough analyses

#### Microbiological analyses

Ten grams of doughs at the end of fermentation time were transferred into 90 mL of sterile physiological solution and homogenized for 2 min in a Stomacher Lab Blender 400 (Seward Ltd, Worthing, West Sussex, UK). Afterwards, decimal dilutions were performed and 100 µL of appropriate dilutions were plated onto MR3i medium for enumeration of LAB and onto MYPG agar containing sodium propionate (2 g/L) for enumeration of *S. cerevisiae*. LAB colonies were counted after incubation for 48–72 h at 30 °C under anaerobic conditions and yeast colonies after incubation for 48 h at 30 °C under aerobic conditions. Plate counts were performed in duplicate.

#### Physico-chemical analyses

The pH and the total titratable acidity (TTA) of the doughs were determined at the beginning and at the end of the fermentation time. The pH values were measured by a pH-meter (Metrohm Italiana Srl, Varese, Italy).

TTA was determined on doughs and *A. platensis* F&M-C256 powder by using 10 g of samples, homogenized with 90 mL of distilled water for 3 min and expressed as the amount (mL) of 0.1 N NaOH needed to reach a pH value of 8.5.

The increase in dough volume was assessed by placing 100 g of each dough in a graduated cylinder (1 L). After 2 h of fermentation at 30 °C the volume of the doughs (in mL) was recorded. The volume increase was calculated using the following formula: (∆V/V0) x 100, where ∆V was the difference between the volume after the 2 h of fermentation and the initial volume (V0)^46^.

### “Crostini” analyses

#### HPLC analysis of organic acids

“Crostini” and *A. platensis* F&M-C256 samples were crushed to powder, diluted ten times and filtered by Amicon® Ultra-4 Centrifugal Filters (3.000 Da NMWL) (Merck Millipore) before the injection. Lactic and acetic acid were determined by High-Performance Liquid Chromatography (HPLC) analysis (Varian Inc, Palo Alto, CA, USA).

#### Free amino acid determination

For the free amino acid (FAA) quantification, water/salt soluble extracts (WSE) of “crostini” were prepared as follows. Five grams of the sample were diluted with 20 mL of 50 mM Tris-HCl (pH 8.8), held at 4 °C for 1 h, vortexed at 15-min intervals, and centrifuged at 20,000 × *g* for 20 min^15^. The supernatants were used for the analysis. Before injection on HPLC for FAA quantification, the sample extracts were prepared according to Galli *et al*.^47^. Separation was obtained with a Kinetex 5 µm C18 100 Å column (150 × 4.60 mm; Phenomenex, Bologna, Italy) connected to fluorimetric detector (Jasco Europe, Lecco, Italy) with wavelengths set at 293 nm (Ex) and 492 nm (Em) under the conditions reported by Tuberoso *et al*.^48^. The quantitative analysis was performed using calibration graphs constructed according to the internal standard method.

#### Proximate chemical composition and colour analyses

The “crostini” biochemical composition was determined as in Abiusi *et al*.^43^. Moisture and ash were analysed following ISTISAN protocols^44^. The total energetic value was calculated following the conversion factors designated in the annex XIV of Regulation (EU) N° 1169/2011^49^.

The colour of “crostini” samples was measured instrumentally using a Minolta CR-400 (Japan) colorimeter with standard illuminant D65 and a visual angle of 2°. The results were expressed in terms of L*, lightness (values increase from 0 to 100%); a*, redness to greenness (+60 to −60 positive to negative values, respectively); b*, yellowness to blueness (+60 to −60 positive to negative values, respectively), according to the CIEL*a*b* system. Chroma, C*_ab_ (saturation), and hue angle, h°_ab_, were also calculated, as defined by: C*_ab_ = [(a*^2^ + b*^2^)]^1/2^; h°_ab_ = arctan (b*/a*). The measurements were conducted under the same light conditions, using a white standard (L* = 94.61, a* = −0.53, b* = 3.62), under artificial fluorescent light at room temperature, replicated twelve times for each formulation sample (one measurement per “crostino”).

#### Phycocyanin, phenolics and antioxidant capacity determination

Phycocyanin content in *A. platensis* F&M-C256 lyophilised biomass and in powdered “crostini” was determined according to Herrera *et al*.^50^. Phycocyanin was extracted suspending freeze dried *A. platensis* F&M-C256 powder or “crostini” powders in a 1% (w/v) CaCl_2_ solution at pH 6.8 (typically 1 g powder in 20 mL of extraction solution). The extracted phycocyanin was spectrophotometrically quantified and purity was assessed at 620 nm and 280 nm using a UV-Vis spectrophotometric reader (Cary 60 UV-Vis, Agilent Technologies, California, USA).

The total phenolic content assay was carried out according to Rajauria *et al*.^51^ using the Folin Ciocalteu assay. Samples of 0.1 g of lyophilised “crostini” were dissolved in 6 mL of deionised water and sonicated for 30 min at the maximum power (frequency 20 kHz, power 130 W) maintaining the temperature below 30 °C by immerging the sample flask in an ice bath (MicrosonTM XL2000, Misonix Inc., Farmingdale, New York, USA). To 100 µL aliquots of each sample, 2 mL of 2% sodium carbonate (Sigma-Aldrich) in water were added. After 2 min, 100 μL of 50% Folin Ciocalteu reagent (Sigma-Aldrich) were added. The reaction mixture was incubated in darkness at 25 °C for 30 min. The absorbance of each sample was measured at 720 nm using a UV-Vis spectrophotometric reader (Cary 60 UV-Vis, Agilent Technologies, California, USA). Results were expressed in gallic acid equivalents (mg GAE g^−1^) through a calibration curve with gallic acid (0 to 150 μg mL^−1^, R^2^ = 0.9907) (Sigma-Aldrich).

To evaluate the radical scavenging capacity of the “crostini” samples, the 2,2-diphenyl-1-picrylhydrazyl (DPPH) radical scavenging assay was carried out according to Rajauria *et al*.^51^. Briefly, the assay was performed by reaction of 1 mL of DPPH radical solution (165 μM, in methanol, Sigma-Aldrich) with 1 mL of sample (0.2 g of lyophilised “crostini” extracted for 30 min in 5 mL of a 1:5 methanol/:water solution). The reaction mixtures were incubated in darkness at 30 °C for 30 min and the absorbances were measured at 517 nm using a UV-Vis spectrophotometric reader (Cary 60 UV-Vis, Agilent Technologies, California, USA). The antioxidant capacity of the samples was expressed as Radical Scavenging Activity (% RSA). Samples RSA capacity was also converted in µg of Vitamin C Equivalent Antioxidant Capacity (VCEAC) per milligram of sample through a calibration curve (ascorbic acid: 0 to 10 µg mL^−1^, R^2^ = 0.9918).

#### *In vitro* digestibility tests

The *in vitro* digestibility was evaluated by the method of Boisen & Fernández^52^, modified by Niccolai *et al*.^32^, on lyophilised “crostini”. The method reproduces the chemical-enzymatic digestion (by gastric and pancreatic juices) that occurs in the proximal tract of the monogastric digestive system. One-gram samples (particle size ≤ 1 mm) were weighed and transferred to 250 mL conical flasks. The analysis comprised two steps of enzymatic digestion: the first performed with porcine pepsin (Applichem, Germany) and the second with porcine pancreatin (Applichem)^20^. The dry matter (IVD) and protein (IVPD) *in vitro* digestibility (%) of *A. platensis* F&M-C256 biomass and of “crostini” samples were calculated from the difference between the initial biomass and the undigested biomass (after correction for the blank assay) divided by the initial biomass and multiplied by 100. Casein (Sigma Aldrich Corp., St. Louis, USA) was utilised as the reference material with 100% digestibility.

#### Sensory analysis

An untrained panel of 35 people, 17 males and 18 females, aged from 18 to 60, evaluated the “crostini” in terms of colour, smell, taste, texture, global appreciation (6 levels from “*very pleasant*” to “*very unpleasant*”). The buying intention was also assessed, from “*would certainly buy*” to “*certainly would not buy*” (5 levels). The assays were conducted in a standardized sensory analysis room, according to the standard EN ISO 8589 (2010).

### Statistical analysis

Statistical analysis of the experimental data was performed using STATISTICA from StatSoft (version 8.0) and using Statgraphics Centurion XV from StatPoint Technologies Inc., through variance analysis (one-way ANOVA), by the Scheffé test – *Post Hoc* Comparison (significance level of 95%, *p* < 0.05) and through ANOVA followed by multiple range tests to determine the least significant differences (LSD) (significance level of 95%, *p* < 0.05). All analyses were conducted at least in triplicate and presented as average ± standard deviation.

## Results and Discussion

### Fermentation parameters of dough and chemical analysis of “crostini” and *A. platensis* F&M-C256

Two common parameters used to characterize the sourdough, Total Titratable Acidity (TTA) and organic acids concentrations, were determined also on *A. platensis* F&M-C256 biomass to evaluate its influence on sourdough characteristics. No data concerning TTA in microalgae biomasses are available in the literature, our results showed a TTA value of 15 mL and lactic acid and acetic acid concentrations of 0.84 g/100 g and 0.42 g/100 g, respectively. Consequently, microalgae addition affected the acidification parameters of doughs and “crostini” as discussed below. Table 3 shows both the values of the principal technological parameters and the cell densities of doughs at the end of the fermentation, and the organic acids and free amino acid (FAA) content in the “crostini”.

**Table 3:** Acidification parameters (pH and Total Titratable Acidity, TTA), volume increase (%, ∆V/V0), microorganism concentrations (LAB and yeast) of the doughs at the end of fermentation and organic acids and free amino acid (FAA) content of “crostini”.

	Doughs							“Crostini”		
	Final pH	-∆ pH	Final TTA (mL)	∆ TTA (mL)	ΔV/V0 x100	LAB (CFU/g)	Yeasts (CFU/g)	Lactic acid (g/100 g)	Acetic acid (g/100 g)	FAA g/100 g dry weight
C	4.66 ± 0.09^a^	0.36 ± 0.05^b^	3.13 ± 0.12^a^	0.70 ± 0.00^a^	196 ± 15^b^	(2.6 ± 0.21) × 10^8a^	(2.7 ± 0.78) × 10^7a^	0.27 ± 0.05^a^	0.01 ± 0.01^a^	0.10 ± 0.02^a^
Ap 2	4.85 ± 0.09^ab^	0.31 ± 0.11^ab^	3.87 ± 0.23^b^	1.70 ± 0.21^ab^	163 ± 21^ab^	(2.6 ± 1.22) × 10^8a^	(3.3 ± 1.37) × 10^7a^	0.29 ± 0.06^a^	0.02 ± 0.01^a^	0.14 ± 0.03^a^
Ap 6	4.90 ± 0.00^ab^	0.17 ± 0.04^a^	5.45 ± 0.21^c^	1.20 ± 0.28^ab^	161 ± 25^ab^	(3.5 ± 0.12) × 10^8a^	(3.9 ± 0.45) × 10^7a^	0.37 ± 0.19^a^	0.05 ± 0.03^b^	0.37 ± 0.01^b^
Ap 10	5.04 ± 0.06^b^	0.17 ± 0.04^a^	6.85 ± 0.21^d^	1.85 ± 0.78^b^	144 ± 15^a^	(3.3 ± 0.47) × 10^8a^	(3.1 ± 0.95) × 10^7a^	0.34 ± 0.11^a^	0.06 ± 0.02^b^	0.45 ± 0.02^c^

(C = control without *A. platensis* F&M-C256 incorporation; Ap 2, Ap 6 and Ap 10 indicate doughs and “crostini” enriched with 2, 6 and 10% (w/w) *A. platensis* F&M-C256 biomass; ∆: difference between the final and the initial value). Results are expressed as average ± standard deviation (n = 3). Different letters in the same column correspond to significant differences (*p* < 0.05).

The control displayed the lowest ∆TTA, 0.70 ± 0.00 mL, and the highest ∆pH, 0.36 ± 0.05 at the end of the fermentation process; the opposite trend was observed for Ap 10, that was characterized by the highest ∆TTA and the lowest ∆pH among the samples. In addition to the acidifying effect, *A. platensis* F&M-C256 acted also as buffering agent, probably due to residual bicarbonates of the growth medium, hence to a higher ∆TTA did not correspond a greater pH decrease. All the samples at least doubled their volume at the end of fermentation time, although the volume increase of Ap 10 was lower compared to the control, indicating that a high percentage of *A. platensis* F&M-C256 negatively affects the gluten network development of the dough. Microorganism concentrations were not affected by *A. platensis* F&M-C256 supplementation, and showed final values of about 8 log CFU/g for *L. farciminis* and 7 log CFU/g for *S. cerevisiae* consistent with those reported for stable sourdoughs^2^. Organic acids concentrations, determined in the “crostini” after cooking, were affected by their content in *A. platensis* F&M-C256 powder rather than by different LAB activity. The amount of lactic acid did not show significant differences among the samples, although a higher lactic acid content was found with the increase of *A. platensis* F&M-C256 concentrations. For acetic acid concentration, a statistically significant (*p* < 0.05) increase was observed for higher *A. platensis* F&M-C256 incorporations (Ap 6 and Ap 10).

FAA content increased with the increasing percentage of *A. platensis* F&M-C256. The addition of 6% or more of microalgal biomass in the recipe significantly (*p* < 0.05) increased the amino acid content of “crostini”.

Table 4 presents the proximate chemical composition of “crostini” incorporated with *A. platensis* F&M-C256 biomass. “Crostini” samples presented 7.4 to 9.3% of moisture, which is in the range of allowed moisture values for this type of dried bakery foods^40^. For Ap 2 “crostini” there is no significant difference in comparison to the control. At or above 6% *A. platensis* F&M-C256 biomass addition there is a significant increase in “crostini” moisture content which might be related to a significantly (*p* < 0.05) higher water absorption capacity of *A. platensis* F&M-C256 biomass in comparison to wheat flour (approximately + 150%)^53^. A significant increase (*p* < 0.05) in ash content was observed for Ap 6 and Ap 10 “crostini” compared to the control (+50% and + 61%, respectively). The higher ash content could be due to the CO_3_^2−^ and HCO_3_^−^ residual in the cyanobacterial biomass. As expected, a higher amount of proteins corresponds to a higher amount of *A. platensis* F&M-C256 biomass in the product. A 44% and 69% increase of protein content between *A. platensis* “crostini” (Ap 6 and Ap 10, respectively) and the control was found. These values were comparable with those found in bread enriched with spirulina^38,39^ and were coherent with theoretical calculation, as also reported by Batista *et al*.^20^ and Bolanho *et al*.^54^ on cookies with 2 and 5% *A. platensis* biomass. As expected, in terms of lipids content, no significant variation (*p* > 0.05) between *A. platensis* F&M-C256 “crostini” (16.5–18.2%) and the control (16.6%) was found. Carbohydrate content of *A. platensis* F&M-C256 “crostini”, in agreement with the cyanobacterial biomass composition, ranged from 57.8 to 59.7% and was not significantly different from the control.

**Table 4:** Proximate chemical composition (g/100 g) of control (without *A. platensis* F&M-C256 incorporation) (C) and of “crostini” enriched with 2% (Ap 2), 6% (Ap 6) and 10% (Ap 10) (w/w) *A. platensis* F&M-C256 biomass.

	Moisture	Ash	Lipid	Protein	Carbohydrate	Energetic value (kcal/100 g)
C	7.7 ± 2.0^a^	1.8 ± 0.3^a^	16.6 ± 0.7^a^	9.9 ± 2.2^a^	61.0 ± 6.7^b^	433.2
Ap 2	7.4 ± 0.4^a^	1.9 ± 0.04^a^	16.5 ± 0.8^a^	10.4 ± 2.4^a^	59.7 ± 4.0^a,b^	429.0
Ap 6	9.1 ± 1.0^b^	2.7 ± 0.1^b^	18.2 ± 0.9^a^	14.3 ± 2.2^b^	57.8 ± 3.8^a,b^	450.5
Ap 10	9.3 ± 0.4^b^	2.9 ± 0.6^b^	16.8 ± 0.8^a^	16.7 ± 1.9^c^	57.8 ± 2.4^a,b^	449.0

Results are expressed as average ± standard deviation (n = 9). Different letters in the same column correspond to significant differences (*p* < 0.05).

Therefore, the newly developed “spirulina crostini” could be regarded as a very interesting protein-fortified bakery product, reaching values as high as 14–17% protein. In fact, for Ap 6 and Ap 10 “crostini”, the protein content corresponds to 12.7 and 14.9%, respectively, of the total energy content of the sample. Therefore, these products can be claimed as *“source of protein”* considering the European Commission Regulation on nutritional claims which states that *“a claim that a food is a source of protein may only be made where at least 12% of the energy value of the food is provided by protein*”^55^.

In terms of energetic content, the values ranged from 429 to 450 kcal/100 g.

### “Crostini” bioactive compounds and antioxidant capacity

The presence of bioactive compounds in *Arthrospira platensis* biomass is associated with its antioxidant potential, immunomodulatory, anti-inflammatory and other activities^56^. Among the main bioactives, *A. platensis* contains phycocyanin, a blue proteic pigment composed of an open chain tetrapyrrole chromophore (phycocyanobilin) covalently attached to an apoprotein, that presents nutraceutical properties and health benefits, principally attributed to its antioxidant activity^57,58^.

**Figure 1:**
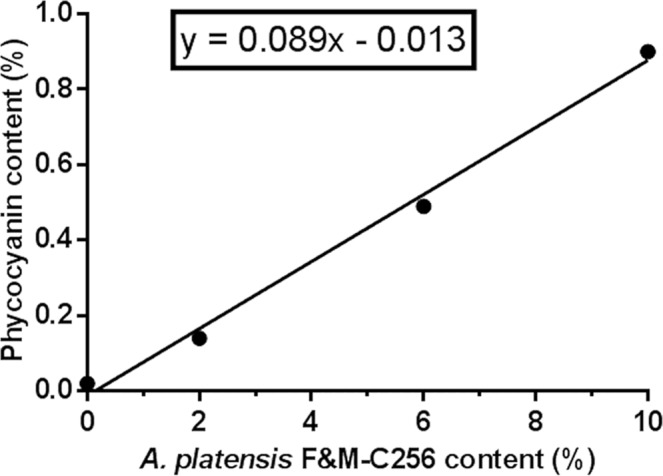
Linear regression between phycocyanin content (%, w/w) of control (without *A. platensis* F&M-C256 incorporation) (0%) and of “crostini” enriched with 2%, 6% and 10% (w/w) *A. platensis* F&M-C256 biomass. Results are expressed as average ± standard deviation (n = 9).

Figure 1 shows the linear regression between phycocyanin content of “crostini” after cooking and level of incorporation of *A. platensis* F&M-C256 biomass. The control “crostini” did not contain phycocyanin. As expected, an increase in the *A. platensis* F&M-C256 biomass content within the “crostini” corresponded to an increase in phycocyanin content. Ap 2, Ap 6 and Ap 10 “crostini” contained 0.1%, 0.5% and 0.9% phycocyanin, respectively. Thus, surpisingly, about all the pigment from the microalgal biomass (which contains 8.2% phycocyanin w/w; results not shown) was still present in the cooked “crostini”. Aliquots of *A. platensis* F&M-C256 biomass (both lyophilised and moistened with 30% water), were heated to simulate the “crostini” baking conditions. The test highlighted an important phycocyanin loss that increased with temperature and exposure time (results not shown). Since phycocyanin reduction during “crostini” baking was much lower compared to that of biomass alone, we hypothesize that some dough components might act as protecting agents against phycocyanin degradation. Further investigation to fully clarify this protective action of dough is necessary.

**Figure 2:**
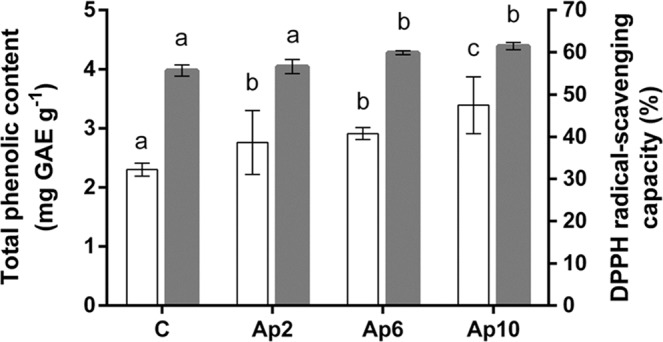
Total phenolic content (expressed as gallic acid equivalents mg g^−1^ dry weight) (white columns) and radical-scavenging capacity (%) (grey columns) of control (without *A. platensis* F&M-C256 incorporation) (C) and of “crostini” enriched with 2% (Ap2), 6% (Ap6) and 10% (Ap10) (w/w) *A. platensis* F&M-C256 biomass. Results are expressed as average ± standard deviation (n = 9). Different letters correspond to significant differences (*p* < 0.05).

Phenolic compounds, synthesized as secondary metabolites by plants, but also by cyanobacteria, are considered one of the most important classes of natural antioxidants^59^. Phenolics, that include simple phenols, flavonoids, phenylpropanoids, tannins, lignins, phenolic acids, and their derivatives^59^, are receiving increasing interest from food manufacturers and consumers for their health benefits^60^. Figure 2 shows the total phenolic content of “crostini”. A value of 2.3 mg GAE g^−1^ was found in the control “crostini”, very likely supplied by the extra-virgin olive oil used in the dough preparation^61^. The addition of *A. platensis* F&M-C256 biomass, with a total phenolic content of 10 mg GAE g^−1^ ± 0.1, resulted in a significant (*p* < 0.05) supplementation of phenolic compounds to the “crostini”. Ap 10 “crostini” presented the highest phenolic content (3.4 mg GAE g^−1^). Other studies reported a higher total phenolic content of *A. platensis* biomass^62^, as well as a correlation with its antioxidant activity^20^. Bolanho *et al*.^54^ reported a total phenolic content for *A. platensis* biomass of 12 mg GAE g^−1^, similar to that found in the present study. They also showed an increase of about 65% (from 1.4 to 2.3 mg GAE g^−1^) in total phenolic content in 5% *A. platensis* cookies when compared to the control, which is higher than the increase found in this study for Ap 6 “crostini” (26%).

The radical-scavenging capacity of *A. platensis* F&M-C256-enriched “crostini” was tested by the DPPH method. The incorporation of *A. platensis* F&M-C256 biomass led to a significant (*p* < 0.05) increase in the radical-scavenging capacity for 6 and 10% *A. platensis* F&M-C256 “crostini” compared to the control (Fig. 2). Overall, DPPH radical scavenging capacity ranged from 57% to 61%, corresponding to a VCEAC of 0.60 to 0.64 µg per mg of “crostini” (from Ap 2 to Ap 10 “crostini”, respectively) (Fig. 2). The control showed a radical scavenging capacity of about 55%, indicating that ingredients in the sourdough preparation already confer a strong antioxidant capacity, that is only slightly increased by *A. platensis* F&M-C256 biomass addition. The results of this work are in agreement with the study conducted by Siriwardhana *et al*.^63^ which reported a high correlation between DPPH radical scavenging activities and total phenolic content. Other authors^64,65^ have studied the antioxidant capacity of *A. platensis* enriched bakery products (principally cookies), attributing this property to the phycobiliproteins provided by this microorganism. El Baky *et al*.^64^ observed increasing antioxidant activity for biscuits containing *A. platensis* biomass (from 0.3 to 0.9%). Singh *et al*.^65^ found a linear positive correlation between *A. platensis* concentration (from 1.6 to 8.4%) in biscuits and antioxidant activity. Our results are in agreement with the findings of El Baky *et al*.^64^ and Singh *et al*.^65^, considering that the antioxidant capacity increases with the increasing levels of *A. platensis* F&M-C256 in “crostini”.

The extra-virgin olive oil used in the dough preparation presumably contributed to the high antioxidant capacity of control “crostini”. The addition of *A. platensis* F&M-C256 biomass, replacing wheat flour in the doughs, weakly increased the radical-scavenging capacity, with a more moderate effect than observed for phenols. After baking, all *A. platensis* F&M-C256 “crostini” still presented a high content of phycocyanin, possibly responsible, together with phenolics, for the observed antioxidant capacity.

### “Crostini” *in vitro* digestibility

The *in vitro* digestibility analysis reproduces the chemical-enzymatic attack that occurs in the proximal tract of the monogastric digestive system through a double enzymatic incubation with pepsin and pancreatin^52^. Most of the literature on algae digestibility focuses on seaweeds^66–68^ and only few studies deal with microalgae^69,70^. This is the first work, to our knowledge, on *in vitro* digestibility of cyanobacteria-enriched “crostini”.

**Figure 3:**
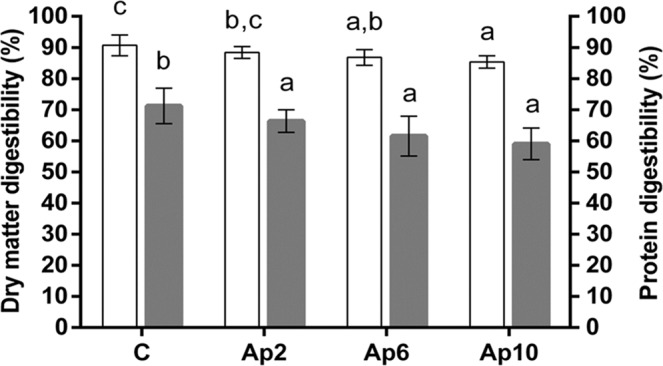
*In vitro* dry matter digestibility (IVD, %) (white columns) and *in vitro* protein digestibility (IVPD, %) (grey columns) of control (without *A. platensis* F&M-C256 incorporation) (C) and of “crostini” enriched with 2% (Ap2), 6% (Ap6) and 10% (Ap10) (w/w) *A. platensis* F&M-C256 biomass. Results are expressed as average ± standard deviation (n = 9). Different letters correspond to significant differences (*p* < 0.05).

The *in vitro* dry matter (IVD) and protein digestibility (IVPD) results are presented in Fig. 3. *A. platensis* F&M-C256 “crostini” showed lower IVD values than the control, but still above 85%. The reason could be that *A. platensis* F&M-C256 biomass is not completely digestible (78% ± 2.6). The dry matter digestibility of Ap 2 and Ap 6 “crostini” (88.4% and 86.8%, respectively) (Fig. 3) was slightly lower compared to the values found for 2 and 6% *A. platensis* F&M-C256-based cookies (94.6% and 92.1%, respectively) by Batista *et al*.^20^, but still within the IVD range of commercial wheat crackers (78.3–93%)^71^.

IVPD values reflect those of dry matter digestibility (Fig. 3). Protein digestibility is an important factor to estimate the availability of protein for intestinal absorption, which influences the efficiency of protein utilization^72^. Many authors reported very diverse protein digestibility values (from 70 to 84%) for different species of *Arthrospira*^73–75^. In our study, an IVPD value of 81% ± 3.0 for *A. platensis* F&M-C256 biomass was found. *A. platensis* F&M-C256 “crostini” showed significantly (*p* < 0.05) lower IVPD values than the control (Fig. 3). This is in accordance with De Marco *et al*.^21^, which evaluated the protein digestibility of *A. platensis* enriched dried pasta (from 5 to 20%). The IVPD reduction at the increasing percentage of *A. platensis* F&M-C256 biomass addition could be due to the content of algae minor compounds (i.e. phenols or polysaccharides) that may react with gluten, forming insoluble complexes and inhibiting the activity of pepsin and pancreatin enzymes^76,77^. However, it should be noted that the control “crostini” has 9.9% total protein (Table 4), with 71% IVPD, which should result in approximately 7 g digestible protein/100 g. On the other hand, the Ap 10 “crostini” has a much higher protein content (16.7%, Table 4), so even with a lower IVPD (59%), there is still a higher digestible protein content, of approximately 10 g/100 g.

### “Crostini” colour and appearance

**Figure 4:**
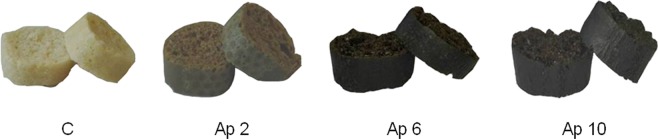
Control “crostini” (without *A. platensis* F&M-C256 incorporation) (C) and “crostini” enriched with 2% (Ap 2), 6% (Ap 6) and 10% (Ap 10) (w/w) *A. platensis* F&M-C256 biomass.

*A. platensis* F&M-C256 “crostini” presented unusual and innovative tonalities. The different samples showed very distinct colours, as evident by visual observation (Fig. 4) and through the instrumental colour parameters analysis represented in Table 5.

**Table 5:** Instrumental color parameters of control (without *A. platensis* F&M-C256 incorporation) (C) and of “crostini” enriched with 2% (Ap 2), 6% (Ap 6) and 10% (Ap 10) (w/w) *A. platensis* F&M-C256 biomass.

	L*	a*	b*	C*	h°
C	69.0 ± 1.0^d^	−1.1 ± 0.1^d^	16.8 ± 0.7^d^	16.8 ± 0.7^d^	93.8 ± 0.2^c^
Ap 2	35.9 ± 0.4^c^	0.7 ± 0.2^c^	12.5 ± 0.6^c^	12.5 ± 0.6^c^	86.6 ± 0.9^b^
Ap 6	28.9 ± 0.8^b^	0.4 ± 0.1^b^	3.7 ± 0.7^b^	3.7 ± 0.7^b^	83.7 ± 1.9^b^
Ap 10	26.7 ± 0.6^a^	0.3 ± 0.1^a^	1.2 ± 0.4^a^	1.2 ± 0.4^a^	76.7 ± 4.3^a^

Results are expressed as average ± standard deviation (n = 12). Different letters in the same column correspond to significant differences (*p* < 0.05).

Regarding luminosity (L*), there was a drastic significant (*p* < 0.05) decrease from the light control sample (69%) to the very dark *A. platensis* F&M-C256 “crostini” samples (36 to 27%). These results are in agreement with previous studies, namely *A. platensis* F&M-C256 cookies^20^ and pasta^78^. In terms of chromaticity (C*), which results from the combination of a* and b* coordinates, it is clear that b* (blue/yellow intensity) parameter is dominant over a* (red/green intensity). In fact, a* values are very close to zero (≤1), which means that green/red colour intensity is very low. On the other hand, the control sample has a b* value of 17, i.e. on the yellow range. By increasing *A. platensis* F&M-C256 concentration, a significant decrease in b* is observed, reaching values close to 1 for the Ap 10 “crostini”. Batista *et al*.^20^ observed a similar behaviour for *A. platensis* F&M-C256 cookies, although with much higher a* and b* values and constant hue angle for cookies with 2% and 6% *A. platensis* F&M-C256 biomass incorporation (h = 117°). In the present study, the hue angle (h°) significantly (*p* < 0.05) decreases with *A. platensis* F&M-C256 concentration. The control sample presents an h° of 94°, which is very close to a pure yellow tonality (90°) while Ap 2 and Ap 6 “crostini” show lower values around 84–87° and Ap 10 “crostini” goes down to 77°. From these results, it seems that *A. platensis* F&M-C256 incorporation is not contributing to a vivid coloration but mainly to a darkening effect. Probably *A. platensis* F&M-C256 pigments, namely green chlorophylls, are highly degraded during the “crostini” high temperature baking and toasting procedures. As will be detailed below (section 3.5) these colour characteristics of the *A. platensis* “crostini” are not well appreciated by the sensory panelists.

The “crostini” characteristic dimensions were also measured (using a digital caliper) and it was observed that the surface area of the “crostini” decreased linearly (R^2^ = 0.96) when increasing *A. platensis* F&M-C256 content (results not shown). In fact, the control “crostini” had a surface area of 10.0 ± 1.6 cm^2^, while, Ap 2, Ap 6 and Ap 10 “crostini” showed surface areas of 8.0 ± 1.4 cm^2^, 5.4 ± 1.1 cm^2^ and 3.9 ± 0.7 cm^2^. This is in agreement with the lower volume increase observed for *A. platensis* F&M-C256 doughs (Table 3). Graça *et al*.^22^ have also reported lower dough volume expansion and lower surface areas on bread enriched with *Chlorella vulgaris*.

### “Crostini” sensory evaluation

**Figure 5:**
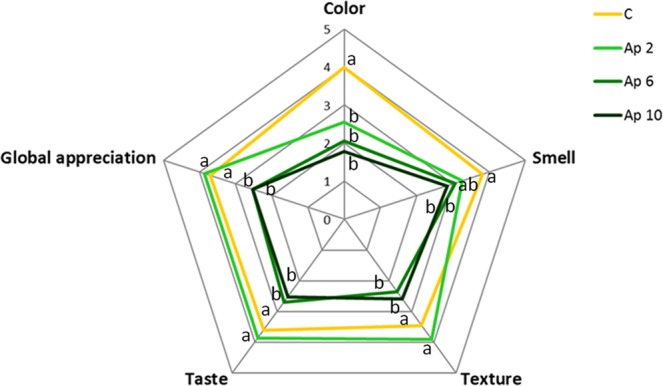
Responses of the sensory analysis panel tasters (n = 35) of control (without *A. platensis* F&M-C256 incorporation) (C) and of “crostini” enriched with 2% (Ap 2), 6% (Ap 6) and 10% (Ap 10) (w/w) *A. platensis* F&M-C256 biomass. 0 – “very unpleasant”; 1 – “unpleasant”; 2 – “slightly unpleasant”; 3 – “slightly pleasant”; 4 – “pleasant”; 5 – “very pleasant”. Different letters correspond to significant differences (*p* < 0.05).

Sensory analysis assays were carried out with *A. platensis* F&M-C256 “crostini”, at 2, 6 and 10% incorporation level. Figure 5 represents the average scores of the sensorial parameters as evaluated by the panel. From the graph it is clear that the less appreciated samples were Ap 6 and Ap 10 “crostini” (average score 2.5 for global appreciation, “slightly unpleasant”) The global sensory appreciation of Ap 2 “crostini” was considered “pleasant” (average score 3.9), similar to the control sample (average score 3.7). In fact, no significant differences (p > 0.05) were found between Ap 2 and control “crostini”, regarding taste, smell or texture attributes, while colour was significantly (p < 0.05) less appreciated (average scores 2.6 and 4.0, respectively). Many authors reported the results of sensory analyses of microalgae-based food products such as bread^38,39,79,80^, pancakes^81^, croissants^82^, cookies^20,54,64,65^ and pasta^83,84^ finding that these products reported different appreciation in terms of sensorial acceptance. Bolanho *et al*.^54^ found that the addition of *A. platensis* biomass (2 and 5%) in the cookies decreased the sensorial acceptance when compared to the control cookie. Singh *et al*.^65^ also reported that the addition of *A. platensis* (>7% incorporation level) to cookies prepared from sorghum and whole wheat flour negatively affected the textural and sensory attributes of flavour. On the contrary, El-Baky *et al*.^64^ found that cookies supplemented with different levels of *A. platensis* biomass (0.3, 0.6 and 0.9% incorporation level) were significantly acceptable for colour, odour, flavour, texture and global appreciation.

**Figure 6:**
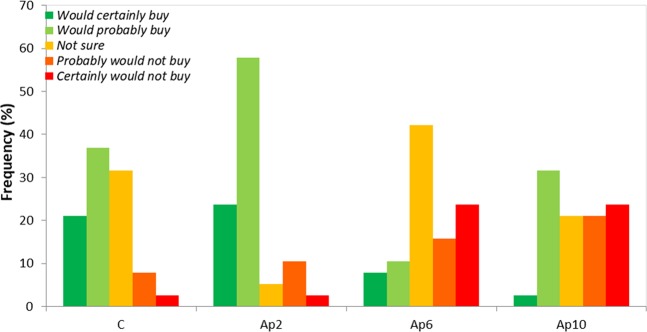
Responses of the sensory analysis panel tasters (n = 35) of control (without *A. platensis* F&M-C256 incorporation) (C) and of “crostini” enriched with 2% (Ap2), 6% (Ap6) and 10% (Ap10) (w/w) *A. platensis* F&M-C256 biomass intention of buying.

Figure 6 presents the answers given by the panelists in relation to the buying intention. Around 60% of the tasters “would probably buy” and 22% “would certainly buy” the Ap 2 “crostini”, the most appreciated product. While, Ap 6 and Ap 10 “crostini” presented a high variability in terms of buying intention. Around 35% of panel tasters would probably buy the Ap 10 “crostini” and less than 15% gave the same response for Ap 6 “crostini”. Maybe Ap 6 “crostini” did not show any distinctive character neither in terms of strong taste nor in terms of delicate taste, as confirmed by insecure tasters (“not sure”, 42%). Around 25% “certainly would not buy” the Ap 6 and Ap 10 “crostini”, principally due to taste and texture (Fig. 6).

## Conclusion

The combination of *Arthrospira platensis* (spirulina) biomass addition and the sourdough technology led to the development of a novel microalgae-based bakery product. The addition of *Arthrospira platensis* F&M-C256 biomass as ingredient resulted in sourdough “crostini” with an innovative green colour. Increasing microalgae content from 2% to 10% (w/w) resulted in a significant (*p* < 0.05) increase in the “crostini” protein, phycocyanin and total phenolic content. Considering the European Commission Regulation on nutritional claims, “crostini” incorporated with 6% and 10% *A. platensis* F&M-C256 can be claimed as *“source of protein”*. A lower value of *in vitro* dry matter and protein digestibility between *A. platensis* F&M-C256 “crostini” and the control was found, although IVD values are always above 85%. Two percent *A. platensis* F&M-C256 “crostini” presented the highest sensory scores and resulted the most appreciated product, even more than the control without cyanobacterial biomass. To improve the acceptance by consumers of 6% and 10% *A. platensis* F&M-C256 “crostini”, educational marketing strategies and formulation enhancements should be considered.

This study suggests that *A. platensis* F&M-C256-based sourdough “crostini” represent original functional food products, with antioxidant properties (also due to the extra virgin olive oil utilization) and containing alternative source of digestible proteins. Moreover, the use of sourdough fermentation in producing *A. platensis* F&M-C256 “crostini” can provide an additional health value considering the enhancing of nutritional properties associated to this biotechnology process. This new product could become widely consumed by particular categories of people such as sportsmen, vegetarians, vegans and the elderly, but also by consumers interested in a product with an innovative taste and colour.
